# 

**Published:** 1905-11-25

**Authors:** 


**Notices of Births, Deaths, and Marriages** are inserted in this column at a charge of 2s. 6d. for an announcement not exceeding four lines.

**MARRIAGE.**

On November 22, at St. James's Church, Piccadilly, by the Rev. J. F. Douglas, Alexander Stewart Hincks, formerly Captain in the 76th Regt., son of the late Sir Francis Hincks, K.C.M.G., C.B., to Ivy Roberta, daughter of the late Robert Warwick Maxwell, of the Manor House, Orton, Waterville, Peterborough. (2332)

**NOTICE TO CORRESPONDENTS.**

All MSS., Letters, Books for Review, and other matters intended for the Editor should be addressed The Editor, "The Hospital," The Hospital Buildings, 28 & 29 Southampton Street, Strand, W.C.

The Editor cannot undertake to return rejected MS., even when accompanied by stamped directed envelope.

**Notice to Advertisers and Subscribers.**

All Advertisements, Orders for Copies of The Hospital, and Business Communications should be addressed to **THE MANAGER (Not to the Editor),** The Hospital, 28 & 29 Southampton Street, Strand, London, W.C.

The Cost of Subscription, post free, is at follows:—Per week, 3½d.; per quarter, 3s. 3d.; per half-year, 6s. 6d.; per annum, 13s. Foreign and Colonial:—Per annum, 19s. 6d., payable, in all cases, in advance.

**NOTICE.—Vol. XXXVII. of "The Hospital" October 1904 to March 1905), in handsome cloth binding, will shortly be on sale at the office of this Journal, price 10s. 6d. Cases for binding the Numbers contained in this Volume 2s. Index to Vol. XXXVII., 3½d., post free.**

**The Monthly Part for November is now ready. Price 1s., by post, 1s. 3d.**

### MEDICAL APPOINTMENTS.

**C. H. Ackland,** L.R.C.P.Lond., M.R.C.S.Eng., L.D.S.Eng., Dental Surgeon to the Royal Victoria Hospital, Bournemouth. **J. H. D. Acland,** M.R.C.S., L.R.C.P., House Surgeon at the East London Hospital for Children, Shadwell. **Herbert William Allan,** B.A.Cantab., L.R.C.P.Lond., M.R.C.S., Medical Officer of Health of Wells (Somerset) and Superintendent of the Isolation Hospital. **James Grant Andrew** M.B., C.M., F.F.P.S.Glasg., Surgeon to the Victoria Infirmary, Glasgow. **H. E. Barrett,** M.R.C.S., L.R.C.P.Lond., Third Anæsthetist to the London Throat Hospital. **E. D. Bascombe,** L.D.S.Eng., Dental Surgeon to the Royal Victoria Hospital, Bournemouth. **Lacey Bathurst,** M.B., B.S.Lond., M.R.C.S. Eng., L.R.C.P.Lond., Senior House Surgeon, Croydon General Hospital. **H. A. L. Birchall,** L.R.C.S.Irel., L.A.H.Irel., District Medical Officer by the Redruth (Cornwall) Board of Guardians. **George Hedwig Carrington,** M.R.C.S., L.S.A. D.P.H.Lond., District Medical Officer by the Poole (Dorset) Board of Guardians. **H. Dove Cormac,** M.B., M.S.Madras, Second Assistant Medical Officer, Cheshire County Asylum, Parkside, near Macclesfield. **R. T. Edwards,** M.R.C.S.Eng., L.R.C.P.Lond., an Examiner to the St. John Ambulance Association. **Laurence Galsworthy,** M.R.C.S., Honorary Anæsthetist to the Italian Hospital, Queen Square, London, W.C. **Lewis Graham,** M.R.C.S.Eng., L.R.C.P.Lond., Junior House Surgeon, Croydon General Hospital. **Robert G. Henderson,** M.A., M.B., Ch.B.Aberd., F.R.C.S.Edin., Honorary Pathologist to the Oldham Infirmary. **Pierce Jones,** M.B., B.S.Glasg., Certifying Surgeon under the Factory and Workshop Act for the Portmadoc District of the Counties of Carnarvon and Merioneth. **Francis James Joynes,** M.R.C.S., L.S.A., reappointed Medical Officer of Health for the Dursley (Gloucestershire) Rural District Council. **J. Kyffin,** L.R.C.P.Lond., M.R.C.S., Certifying Surgeon under the Factory and Workshop Act for the Gosport District of the County of Hants. **Sydney A. Owen,** B.A., M.B., B.C.Cantab., M.R.C.S., L.R.C.P., Resident Medical Officer at the East London Hospital for Children, Shadwell. **Charles Wightwick Pitt,** M.R.C.S., L.S.A., reappointed Medical Officer of Health of Malmesbury (Wilts). **John Llewellyn Prichard,** L.R.C.P.Lond., M.R.C.S., Assistant Medical Officer at the Joint Counties Asylum, Carmarthen. **W. M. Robson,** M.D.Lond., M.R.C.P. Lond., Honorary Assistant Physician to the General Hospital, Northampton. **F. H. Thiele,** M.D.Lond., reappointed Pathologist to University College Hospital, London. **Herbert Tilley,** B.S., F.R.C.S., Surgeon to the Ear and Throat Department of University College Hospital, London.

### HOW TO BECOME A NURSE

**Full particulars of Training Schools, Premiums, Salaries, Hours of Duty, and all other details required by anyone desirous of entering the Nursing Profession will be found in**

**THE NURSING PROFESSION: How and Where to Train.**

By Sir HENRY BURDETT, K.C.B.

Price 2/- net, 2/4 post free.

**It is essential that anyone desirous of taking up Nursing should have some idea of the duties of a Nurse.**

**NURSING HINTS TO PROBATIONERS ON PRACTICAL WORK.**

By ANNESLEY VOYSEY. Price 2/- net, 2/4 post free.

**Contains full particulars of a Nurse's duties and will prove of the greatest assistance to the Probationer.**

### Some Books indispensable to the Nurse Probationer

Dictionary of Terms used in Medicine, Midwifery and Nursing.

**THE NURSES' PRONOUNCING DICTIONARY.**

By HONNOR MORTEN. New Edition, Revised by MARY I. BURDETT. 2/- post free.

This Dictionary has been used by Nurses for many years, and the fact that it has been recently revised and the phonetic pronunciations added by Miss M. I. Burdett should greatly enhance its value as a work of reference. Its size enables it to be carried comfortably in the apron pocket, which is a strong point in its favour.

How to Bandage.

**PRACTICAL GUIDE TO SURGICAL BANDAGING AND DRESSINGS.**

By WM. JOHNSON SMITH, F.R.C.S. 2/- post free.

A useful guide to Bandaging and Dressing, written especially for Nurses by a surgeon of great experience. The work is up to date and the information thoroughly reliable.

Anatomy and Physiology.

**ELEMENTARY ANATOMY AND SURGERY FOR NURSES.**

By WILLIAM McADAM ECCLES, M.S.Lond., M.B., F.R.C.S., L.R.C.P. 2/6 post free.

**ELEMENTARY PHYSIOLOGY FOR NURSES.**

By C. F. MARSHALL, M.D., B.Sc., F.R.C.S. Crown 8vo, Illustrated, cloth. 2/- post free.

A valuable help to Physiology for Probationers.

Medical and Surgical Nursing.

**A HANDBOOK FOR NURSES.**

By J. K. WATSON, M.D., M.B., C.M. New and Revised Edition. Profusely Illustrated. 5/- net, 5/4 post free.

Medical Electricity.

**MEDICAL ELECTRICITY AND THE LIGHT TREATMENT.**

By KATE NEALE, Sister in Charge Light Department, Guy's Hospital. Cloth, profusely Illustrated. 2/6 net, 2/9 post free.

A practical book on a subject hitherto untouched from a nursing standpoint.

A Complete Text-Book of Medical, Surgical, and Monthly Nursing.

**NURSING: ITS THEORY AND PRACTICE.**

By PERCY G. LEWIS, M.D., M.R.C.S., L.S.A. New Edition. Profusely Illustrated. 3/6 post free.

Surgical Work.

**SURGICAL WARD-WORK AND NURSING.**

By ALEXANDER MILES, M.D.Edin., C.M., F.R.C.S.E. Second Edition. With particulars and Illustrations of the Instruments used in various Operations. 3/6 net, 3/10 post free.

**SURGICAL INSTRUMENTS AND APPLIANCES USED IN OPERATIONS.**

A Classified and Illustrated List, with Explanatory Notes. By HAROLD BURROWS, F.R.C.S. Ready Shortly. Limp cloth. 1/6 net, 1/8 post free.

**This work will prove of the utmost service as a work of reference to all Nurses, as not only does it contain particulars of instruments but is fully illustrated so that they can be readily recognised.**

**THE EXAMINATION OF THE URINE.**

Compiled for the use of Nurses. By J. K. WATSON, M.D., M.B., C.M. 1/- net, 1/1 post free.

***Write for Complete Catalogue which will be sent post free on application.***

**The Scientific Press, Ltd.**

**Publishers of Nursing Text-Books, Charts, and Case-Books of all Descriptions.**

**28 & 29 SOUTHAMPTON STREET, STRAND, LONDON, W.C.**

